# Application of Response Surface Methodology to Design and Optimize Purification of Acetone or Aqueous Acetone Extracts of Hop Cones (*Humulus lupulus* L.) Using Superparamagnetic Iron Oxide Nanoparticles for Xanthohumol Isolation

**DOI:** 10.3390/ma17194827

**Published:** 2024-09-30

**Authors:** Natalia Żuk, Sylwia Pasieczna-Patkowska, Jolanta Flieger

**Affiliations:** 1 Department of Analytical Chemistry, Medical University of Lublin, Chodźki 4A, 20-093 Lublin, Poland; natalia.zuk@umlub.pl; 2 Faculty of Chemistry, Department of Chemical Technology, Maria Curie-Skłodowska University, Pl. Maria Curie-Skłodowskiej3, 20-031 Lublin, Poland; sylwia.pasieczna-patkowska@mail.umcs.pl

**Keywords:** iron oxide nanoparticles, *Humulus lupulus* L., hop cone extracts, acetone/water hop extract, acetone hop extract, magnetic dispersion extraction

## Abstract

Iron oxide nanoparticles (IONPs) are an ideal sorbent for magnetic dispersion extraction due to their superparamagnetic properties and developed and active surface. This work aims to use IONPs, obtained by chemical co-precipitation, to purify 100% acetone and 50% acetone extracts from hop cones (*Humulus lupulus* L.) obtained by ultrasonic-assisted solvent extraction. The extracts were purified from bitter acids (i.e., humulones, lupulones) to isolate xanthohumol. The sorption conditions were optimized depending on the composition of the extraction mixture, specifically the mass of IONPs and the time needed to achieve effective sorption using response surface methodology (RSM). An analysis of variance (ANOVA) was performed to assess the adequacy of the developed model, and a good agreement was found between the experimental data and the proposed model. The polynomial equation describing the model is highly significant (*p* < 0.05), with a precision of Adeq (above 4). This indicates the usefulness of the polynomial regression model for prediction in experimental design. The final products of the purification for 100% acetone extracts and 50% acetone contain 40.58 ± 2.84 µg mL^−1^ and 57.64 ± 0.83 µg mL^−1^ of xanthohumol, respectively. The use of 50% acetone extract provides more favorable conditions due to the smaller amount of nanoparticles required for extract purification and a higher recovery of xanthohumol. The development of a reliable multivariate model allowed for the optimization of the extract purification process, resulting in high-purity xanthohumol from natural sources.

## 1. Introduction

Hops (*Humulus lupulus* L., *Cannabaceae*) contain numerous secondary metabolites, primarily prenylated flavonoids such as xanthohumol (XN), its isomer isoxanthohumol (IXN), and desmethylxanthohumol (DMXN). They also contain derivatives such as 8-prenylnaringenin (8-PN) and 6-prenylnaringenin (6-PN), bitter acids (humulones and lupulones), and essential oils [1,2]. In recent years, there has been growing interest in xanthohumol, a prenylated flavonoid found in the female inflorescences of the hop plant, due to its numerous health-promoting properties, including its anticancer, antidiabetic, strong antioxidant, anti-inflammatory, antibacterial, and immunomodulatory effects [3,4,5,6,7,8,9]. Wang et al. [10] recently confirmed the neuroprotective properties of xanthohumol in Alzheimer’s disease, which acts against the aggregation of β-amyloid. Cancer, diabetes, and Alzheimer’s are diseases that pose a serious threat to health. The global number of people with various forms of dementia, diabetes, and cancer is growing with the development of civilization. The incidence of Alzheimer’s disease and other forms of dementia in the world was 43.8 million in 2016. A further increase in the number of cases is predicted, which, in 2050, will amount to over 150 million according to Dementia Forecasting Collaborators, 2022 [11,12]. The International Agency for Research on Cancer (IARC) has released estimates that the incidence of cancer is rising, with 20 million new cases reported in 150 countries in 2022 alone [13]. The situation is alarming in the case of metabolic diseases such as diabetes and obesity. In 2021, there were 529 million people with diabetes [14].

Despite numerous studies confirming xanthohumol’s beneficial pharmacological effects and the availability of many dietary supplements and nutritional products containing xanthohumol on the market, it has not yet been approved as a drug by the US Food and Drug Administration (FDA) [15]. The recommended daily intake of xanthohumol as a dietary supplement ranges from 1.35 to 2.5 mg kg^−1^ per day [16].

Xanthohumol is present in beer at concentrations below 0.1 mg L^−1^. However, the amount can vary depending on the type of beer [17]. In specially enriched beers, the concentration of xanthohumol can range from 1.9 mg L^−1^ to 10 mg L^−1^ [18]. Several methods of xanthohumol extraction have been developed, both from hop cones and the by-products that remain after the extraction of hops with supercritical carbon dioxide [7,19,20,21]. An alternative method of obtaining xanthohumol is microbial biosynthesis using yeast, e.g., *Saccharomyces cerevisiae* or *Escherichia coli* bacteria [22,23]. Methods using a multi-stage chemical synthesis of xanthohumol have also been proposed, e.g., from floracetophenone [24] or naringenin [25].

The extraction of xanthohumol from hop cones is usually multi-step and first includes solvent extraction using chloroform, methylene chloride, methanol, diethyl ether, acetone, and hexane, followed by purification [26]. In order to improve the extraction efficiency, extraction methods assisted by ultrasound, microwaves, and pressurized liquid extraction (PLE) [27,28] as well as ecological approaches, reducing the consumption of organic solvents, such as deep eutectic solvents (DES)-based procedures [17,29], are also used. Macchioni et al. [30] developed conditions for xanthohumol extraction using lactic acid-based deep eutectic solvents (LaDES), i.e., lactic acid/sucrose, achieving a very good extraction efficiency of about 1161 µg g^−1^. An example of an extraction procedure is the one proposed by Chen et al. [20]. The authors of the study used reflux extraction with ethanol at 40 °C in the first stage, followed by isolation using countercurrent chromatography in a two-phase system of n-hexane–ethyl acetate–methanol–water, which provided a yield of 93.6% and a purity of 95.8%.

There is a need to develop new ecological, yet fast and cheap methods that will ensure the efficient purification of rich plant extracts in order to isolate the desired substances. Traditional methods, i.e., solvent extraction and SPE use volatile, toxic organic solvents and are time-consuming. Methods are needed that will not only meet the requirements of green chemistry, but will be (i) fast, i.e., will provide fast mass transfer and the procedure will not require many steps, (ii) will be cheap from the point of view of energy consumption and equipment needs, (iii) will not be labor-intensive (iv), and (v) will not cause too much loss. Only such methods have a chance to find industrial application on a larger scale.

Magnetic iron oxide nanoparticles exhibit a number of advantageous properties as sorbents, namely a large surface-to-volume ratio, fast kinetics, strong adsorption abilities, a highly reactive surface, and magnetism. Iron oxide nanoparticles can be used both as nanoadsorbents and as the magnetic core in core–shell nanosorbents. Magnetic nanomaterials can interact with animal tissues and be involved in biological systems, i.e., magnetotactic bacteria and cryptochrome flavoproteins. Winkler et al. [31] described, in a review article, their contribution to the development of quantum technologies. Iron nanoparticles are considered a relatively new technology in biotechnological and environmental remediation. Their excellent properties of capturing micro- and nanoplastics from aqueous sources are exploited [32]. An important advantage of iron oxide nanoparticles such as Fe_3_O_4_ is the fact that they have strong magnetic properties, relatively low cytotoxicity, and better biocompatibility and biodegradability compared to other nanoparticle materials. Many studies have confirmed that iron nanoparticles are able to immobilize heavy metals and reduce organic and microbial contamination (e.g., bacteria, chlorinated organic compounds, dyes, radionuclides, viruses) [33,34]. Iron oxide nanoparticles (Fe_2_O_3_ or Fe_3_O_4_) obtained by co-precipitation were characterized in terms of shape, size, surface structure, and magnetic properties in previous works [9,35,36]. It was found that iron oxide nanoparticles range in size from 80 to 140 nmn, are mesoporous with a surface area (BET) of 151.4 m^2^ g^−1^, and are characterized by the presence of hydrated iron oxide FeO(OH) on the surface. Magnetic nanoparticles are used as sorbents for magnetic dispersive micro-solid phase extraction (MSPE). This technique uses an external magnetic field to easily and quickly separate the solid adsorption material from the liquid sample matrix [37,38].

Considering the bioactivity of xanthohumol, cheap and efficient methods of its extraction from natural sources are sought. Since solvent extraction methods are not selective, it is desirable to develop methods for their effective purification. There is still a lack of highly selective, fast, cheap, and environmentally friendly methods for obtaining active substances from plant extracts, including xanthohumol, which is obtained on a large scale for the cosmetics, dietary supplements, and pharmaceutical industries. The main goal of our research was to develop a method for purifying hop cone extracts using cheap approaches and in a minimum number of steps. To our knowledge, the use of magnetic nanoparticles for this purpose has not been studied before. We tested the type of solvent and the possibility of adding water to increase XN recovery. The response surface methodology (RSM) approach was applied to evaluate the effect of process parameters, such as the mass of nanoparticles and contact time, and determine the optimal conditions for chosen systems. RSM is a multivariate statistical tool that offers an approach to study the sorption process. RSM provides better repeatability of results and process optimization with a good perspective for the development of a predictive model. In RSM, response surfaces are a graphical representation used to describe the effects of process variables and their consequences on the response. Central composite designs (CCD) and Box–Behnken design (BBD) are two main factorial designs used to evaluate the quadratic response surface and to develop second-order polynomial models in RSM [39,40,41]. CCD is a fractional factorial design, while BBD is a spherical, three-level fractional factorial design, consisting of a center point and midpoints of the edges of a circle circumscribing a sphere. RSM has been previously successfully used as an optimization tool that facilitates the identification of interdependencies between variables in the extraction process from plant materials [42,43,44,45,46]. The application of RSM to find the optimal conditions should be preceded by the analysis of the process parameters using, for example, the Taguchi, Plackett–Burman, and other methods. There are many reports illustrating the application of RSM in practice [47,48,49,50,51,52,53].

In this study, CCD was used to evaluate the effect of process parameters on the response. In RSM, the optimization of process variables involves seven different steps. These steps include (i) the selection of the response (alpha and beta acid removal efficiency), (ii) selection of variables and assigning codes to them, (iii) development of the experimental design for the removal of contaminants, (iv) regression analysis, (v) generation of quadratic polynomial, i.e., response development, (vi) development of a two-dimensional contour plot or three-dimensional surface of the observed response surface, and finally (vii) analysis of the optimum operating conditions. The optimized experimental conditions with the highest efficiency were applied, and finally, the identity of the isolated xanthohumol was confirmed by FT-IR.

## 2. Materials and Methods

### 2.1. Materials

Acetone and acetonitrile were purchased from E.Merck (Darmstadt, Germany). Standard xanthohumol was obtained from Sigma-Aldrich (St. Louis, MO, USA) (≥96% HPLC purity). Water with a resistivity of 18.2 MΩ cm was obtained from an ULTRAPURE Millipore Direct-Q 3UV-R (Merck, Darmstadt, Germany). Hop cones (*Humulus lupulus*) of the Marynka variety were grown in the region of southeastern Poland (Chmielnik, 20°45′ E 50°37′ N) and harvested in September 2023. Hop cones EKO (*Humulus lupulus*—strobili) produced by Dary Natury (Grodzisk, Poland) is an ecological product from Podlasie in northeastern Poland. Hop pellets “Lubelski” produced by NB Minerals (Tychy, Poland).

### 2.2. Synthesis of Iron Oxide Nanoparticles (IONPs)

The IONPs were obtained by the co-precipitation method, which was described previously [9,33,34]. In brief, to a mixture of iron salts in 2:1 molar ratio of Fe(III) to Fe(II), ammonia aqueous solution was added dropwise with constant stirring at a room temperature. The black precipitate was separated, washed with deionized water, and dried. The nanoparticles obtained by the co-precipitation method were characterized by scanning electron microscopy (SEM), energy dispersive spectrometry (EDS), X-ray photoelectron spectroscopy (XPS), and Brunauer–Emmett–Teller technique (BET), DLS, the magnetic behavior, and superparamagnetism of the obtained nanoparticles were measured by the hysteresis loop using a magnetometer in the previous works [9,35,36].

### 2.3. HPLC-DAD

The HPLC-DAD system VWR/Hitachi LaChrom Elite (Merck, Rahway, NJ, USA) equipped with the LaChrom Elite L-2455 DAD detector, a manual injection valve, and a Jetstream Plus 2 Column Ovens HPLC Heater/Cooler—5480 was used. The analysis conditions were adapted from previous work [54]. Mobile phase at gradient elution mode consisting of 0.1% formic acid in water (*v*/*v*) (A); acetonitrile 0.1% formic acid (*v*/*v*) (B); gradient elution: 20% B, 0–3 min; 20%–50% B, 3–6 min; 70% B, 6–15 min; 100% B, 15–20 min; 100% B, 20–25 min; 100%–20% B, flow rate 0.5 mL min^−1^; and the stationary phase, a Zorbax Extend C18 Agilent Technologies (Santa Clara, CA, USA) column (150 mm × 4.6 mm I.D., 5 μm) were utilized for chromatographic analysis. UV–vis spectra were measured at 200–800 nm. Detection of xanthohumol was performed at a wavelength of 369 nm. Detection of alpha and beta acids peaks’ area was measured at 237 nm. The flow rate was 0.5 mL min^−1^, the column temperature was 25 °C, and the injection volume was 20 μL.

### 2.4. Quantitative Determination of Xanthohumol by HPLC-DAD Method

Quantitative determinations were performed by the external standard method based on a calibration curve. Ten calibrators ranging from 0.12 µg/mL to 160 µg/mL were used to construct calibration curve determined by plotting the peak area (y) versus the concentrations of the calibrators (x):

xanthohumol peak area = 759182.18 (±15595.74) × xanthohumol concentration [µg mL^−1^] + 448346.21 (±341685.46), R^2^ = 0.9966; s_e_ = 904393; *n* = 8; F = 2369.63 (1)

The correlation coefficient (R^2^) was higher than 0.99, indicating the good linearity and reliability in quantification. Xanthohumol was analyzed three times for each concentration level. The precision of the chromatographic method was assessed by repeatedly injecting several levels within the standard curve range within a day for repeatability and between days for intermediate precision, and percentage relative standard deviation (RSD < 5%) values were determined for all data. The limits of detection (LOD = 0.02 µg/mL) and quantification (LOQ = 0.07 µg/mL) were determined experimentally considering a signal-to-noise ratio of 3 and 10, respectively.

### 2.5. Extraction of Spent Hops

The hops were extracted with acetone or 50% acetone/water (*v*/*v*). For the extraction, 2.5 g of dried plant material was suspended in 50 mL of solvent and sonicated for 60 min in an ultrasonic bath (ultrasonic power 1200 W, frequency 35 kHz), a Bandelin Sonorex RK 103 H (Bandelin Electronics, Berlin, Germany). The temperature did not exceed 22 °C thanks to the addition of ice. Then, the extracts were filtered through Whatman No. 1 filter paper using a vacuum filtration apparatus. The extracts were filtered again by the use of Kinesis KX Syringe Filter, Nylon, 13 mm dia., 0.22 µm (St Neots, Cambridgeshire, UK) before HPLC analysis. 

### 2.6. Magnetic Dispersive Micro-Solid Phase Extraction (MSPE)

The IONPs from 0 to 800 mg were added to 1 mL of extract and placed in a rotor at maximum speed (30 RPM) at room temperature. The phases were then separated by applying an external magnet. After phase separation, the upper liquid phase was analyzed chromatographically.

### 2.7. Conductivity Measurements

The kinetics of ion release from IONP in aqueous, acetone and 50% acetone suspensions were monitored by conductivity measurement. The limiting molar conductivity of Fe3+ ions is Λ0 = 204 S cm^2^ mol^−1^, so their potential release can be expected to affect the measurement. The change in conductivity of dispersions containing 700 mg IONP and 3 mL of the corresponding extract was measured over time. In pure water, a slight increase in conductivity (G) of 4.98 µS was observed, while for 100% acetone and 50% acetone extract, the conductivity decreased by up to 0.13 ± 0.09 mS and 0.32 ± 0.24 mS with time, respectively. Conductivity was measured using a multifunctional pH/ORP/conductivity meter CRC-461 Elmetron (Zabrze, Poland) with a sensor ECF-1 Elmetron (Zabrze, Poland). Conductivity was measured in ambient conditions at time intervals of 0, 2, 7, 24, and 31 h, respectively.

### 2.8. FT-IR/ATR Analysis

Attenuated total reflectance Fourier-transform infrared (FT-IR/ATR) spectra of samples were obtained by scanning with spectral resolution of 4 cm^−1^ between 3800 and 500 cm^−1^, at room temperature using Nicolet 6700 spectrometer (Thermo Fisher Scientific, Waltham, MA, USA) and Meridian Diamond ATR accessory (Harrick Scientific Products, Inc., Pleasantville, NY, USA). Liquid samples were applied directly onto the ATR crystal and air-dried until the solvent evaporated. The use of mild drying conditions ensured that no decomposition of the xanthohumol occurred. Each spectrum consisted of 256 scans. Dry potassium bromide (48 h, 105 °C) was used to collect background spectrum. All spectra were corrected for water vapor and carbon dioxide, and ATR correction was applied. No smoothing functions were applied. All spectral measurements were performed at least in triplicate. Raw spectra were processed using OMNICTM software (Thermo Fisher Scientific Inc., Waltham, MA, USA) version 8.2.387.

### 2.9. RSM Analysis

Sequential experiments were conducted to develop the process. Central composite designs (CCD) were used to estimate the quadratic response surface and to develop second-order polynomial models. Process optimization includes estimating coefficients, predicting the response, and checking the acceptability of the developed model. The response is represented by Equation (2):

*Y* = f(*x*_1_, *x*_2_,……,*x_n_*) + *E* (2)

where *Y* is the response, f is the response function, *x*_1_…*x_n_* are independent variables, and *E* is the experimental error. The response function (f) depends largely on the nature of the relationship between the response and the independent variables. The quadratic polynomial model is represented by Equation (3):

(3) $$Y=\beta_{0}+\sum_{i=1}^{k}\beta_{i\;}x_{i}+\sum_{i=1}^{k}\beta_{ii\;}x_{i}^{2}+\sum_{i<j}\sum \beta_{ij}x_{i}x_{j}+E$$

where *Y* is the predicted response; *β*_0_ represents the intercept or regression coefficient; *β_i_, β_ii_,* and *β_ij_* represent the linear, quadratic, and interaction coefficients; *x_i_* and *x_j_* are the coded values of the process variables; and *E* is the experimental/residual error [41].

### 2.10. Statistical Analysis

RSM was developed using TIBC Statistica 13.3, 1984–2017-TIBCO software Inc. to optimize two different independent process variables, i.e., IONPs mass and contact time, and also to evaluate the effect of these variables on the dependent variable, i.e., alpha and beta acid removal efficiency. The effects of each numerical factor was varied at 3 levels, i.e., low, medium, and high. ANOVA analysis was used to confirm the validity of the model. The established conditions were finally checked experimentally. Statistically significancy was established as *p* ≤ 0.05 with a 95% confidence level. Experiments were repeated at least three times and presented as mean ± standard deviation.

## 3. Results

### 3.1. Comparison of Xanthohumol Content in Acetone Extracts Prepared from Different Varieties of Hops

**Figure 1:**
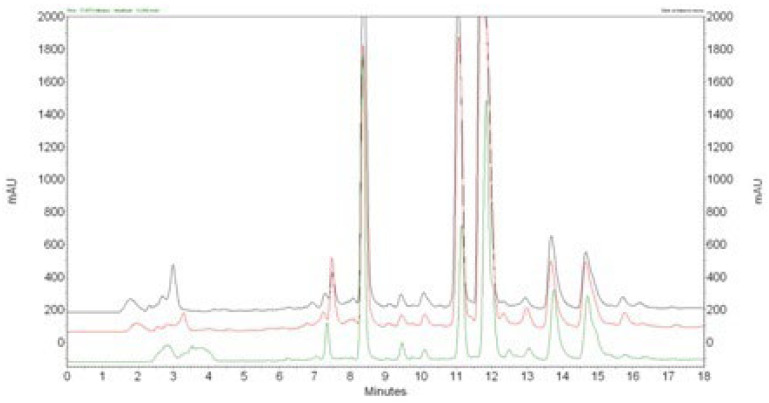
HPLC chromatograms monitored at 369 nm for xanthohumol (tr = 8.3 min) determination in different hop varieties: (green) “Lubelski”; (red) “Marynka”; (black) “Dary Natury”.

The chromatograms obtained for extracts of different hop varieties prepared using 100% acetone are shown in Figure 1. All the tested extracts had similar chemical composition profiles. The main component of all extracts is xanthohumol, which was identified based on identical retention times and UV spectra with the standard. The extracts also show the presence of soft resins with longer retention times and several peaks with shorter retention times compared to xanthohumol. Soft resins mainly consist of bitter acids (alpha and beta), which give beer its characteristic bitterness. Their content is influenced by the type of hops used. The peak of xanthohumol is distinctly separated from other peaks in the chromatogram. The suggested chromatographic system, using gradient elution, ensures the appropriate retention time (tr = 8.4 min), which is an asymmetry factor (As = 0.94).

The content of xanthohumol in extracts, prepared from different varieties of hop cones, is presented in Table 1. In the case of “Dary Natury”, the content of xanthohumol was the highest and exceeded 100 µg/mL of extract. The content of xanthohumol was less satisfactory for “Marynka” and “Lubelski”. The “Dary Natury” variety was selected for further study for this reason.

### 3.2. Sorption Capacity of IONPs in Relation to Hop Cones 100% Acetone Extract

The efficiency of extracting and purifying extracts using nanoparticles depends on the physical and chemical properties of the extractants, including their acid–base properties, dipole moments, surface tensions, and wetting angles. These properties affect the ability to penetrate and wet solid surfaces. Conventional solvents such as alcohols (methanol and ethanol), acetone, diethyl ether, or ethyl acetate are typically used for extraction. In this study, acetone was chosen for its versatility, as it has both polar (C=O) and nonpolar (C-CH_3_) properties. Additional purification steps are often required to isolate xanthohumol from the obtained extracts, which can be time-consuming and increase the overall cost of the process.

**Figure 2:**
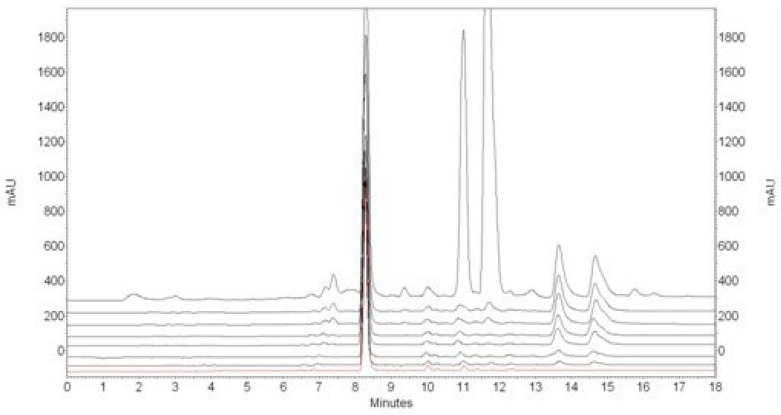
**Overlaid** HPLC chromatograms recorded after 0 h, 1 h, 2 h, 4 h, 6 h, 7.5 h., 9.5 H, 22 h, 24 h (lines from top to bottom) after mixing 1 mL of acetone extract of hop cones “Dary Natury” and 700 mg of IONPs.

**Figure 3:**
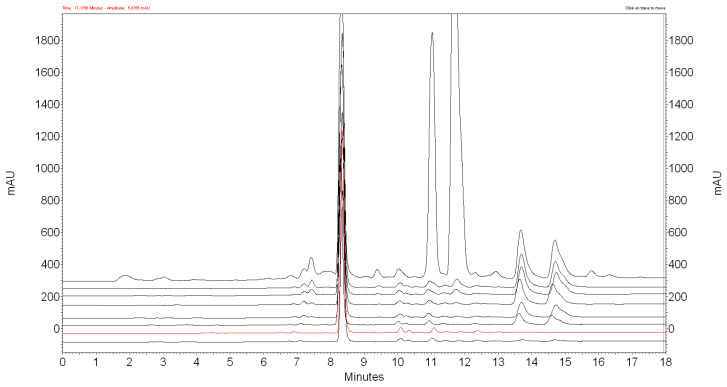
HPLC chromatograms recorded 24 h after mixing 1 mL of acetone extract of hop cones “Dary Natury” with 0, 200, 300, 350, 400, 500, 600, 700, 800 mg of IONPs (from top to bottom).

The IONPs remove alpha acids from the acetone extract at a first-order rate. Beta acids can be removed by increasing the contact time (see Figure 2) or by increasing the amount of nanoparticles (see Figure 3). However, along with alpha and beta acids, the nanoparticles also partially remove xanthohumol from the extract. After 700 mg of IONPs come into contact with 1 mL of acetone extract for 24 h, about 60% of xanthohumol is lost. The concentration of xanthohumol in the purified extract is 40.59 ± 2.84 µg/mL.

### 3.3. The Evaluation of the Optimal Conditions for Extract Purification Using Response Surface Methodology (RSM)

In order to determine the optimal conditions for alpha and beta acid removal, an approach based on the RSM was used. The independent variables were the IONPs mass and the IONPs contact time with the extract. Independently, RSM analysis was performed for 100% acetone and 50% acetone extracts. Before performing the RSM analysis using the Plackett–Burman Design (PBD), we investigated whether selected factors significantly affected the acid removal process from extracts [55]. We then used RSM to determine the optimal range for the relevant factors, with the goal of maximizing alpha and beta acid removal efficiency and reducing time, solvents, and waste.

Using Plackett–Burman (PBD), the simultaneous effects of two studied factors involved in the sorption process (IONPs mass and contact time) were tested to understand their relative effects on the sorption efficiency. Each independent variable was evaluated at two levels: −1 for low level and +1 for high level (Table 2) and were screened by conducting eight experiments. The PBD results confirmed the significant effects of the studied factors, which were then verified in a full factorial experiment. The experimental design of PBD (factors and tested range) is presented in Table 3.

The statistical analysis of the model was performed using the ANOVA test (Table 4 and Table 5). The analysis includes Fisher’s test (F test), its associated probability P (F), and the coefficient of determination (R^2^), which measure the goodness of fit of the regression model.

The observed R^2^ values showed the adequacy of the model. The adjusted R^2^ value in this study shows the high significance of the model. *p* values less than 0.05 indicate that the selected variables of the model are significant.

**Figure 4:**
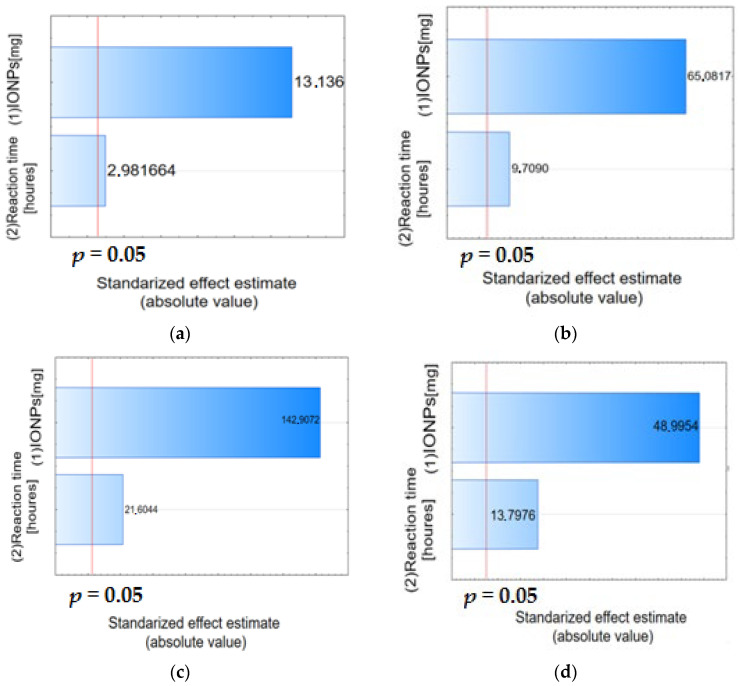
Pareto chart of standardized effects, variable alpha acid (**a**) and beta acid (**b**) removal efficiency [%] in 100% acetone and alpha acid (**c**), and beta acid (**d**) removal efficiency [%] in 50% acetone.

The Pareto chart can also be used to identify significant factors. As can be seen in Figure 4, the parameters examined have a confidence level greater than 95% and can be considered significant.

#### 3.3.1. Sorption Capacity of IONPs to Alpha and Beta Acids from 100% Acetone Hop Cones Extract

The experiments were designed for reaction time from 20 to 30 h and IONPs mass from 650 to 800 mg. Each numerical factor was varied at three levels, i.e., low, medium and high. Table 6 shows the actual values of the factors and their corresponding coded levels. In order to develop the quadratic model, experiments were conducted for at least three levels of each factor, and the levels were evenly spaced. A total of 13 experimental runs were generated. According to RSM, 13 experiments were conducted and their response variable, i.e., α acid removal efficiency, was evaluated (Table 7).

The mathematical model generated by the RSM approach was validated by conducting an experiment on a given optimal system and tested by ANOVA analysis using various statistical parameters (Tables S1 and S2).

The ANOVA analysis of the model suggests that the independent variables had a significant effect (*p* < 0.05) on the prediction of the response (acid removal efficiency). Routinely, the coefficient of determination (R^2^) is used to assess the adequacy of the model. Based on the ANOVA results, the R^2^ for alpha acid removal is 0.9860, whereas for beta acids, it is 0.9963. In addition to the R^2^ coefficient, a useful statistical tool to assess the adequacy of the model is the adjusted R^2^, which in the case of alpha and beta acids is 0.9760 and 0.9936, respectively. In both cases the adjusted R^2^ is close to R^2^, which indicates the adequacy of the constructed model to predict the process response [51]. The lack of fit test provides a measure of the variability of the measurement results for each unique combination of input quantity values. This measure serves as an indicator of the random error in the measurements. It is a test of the residual variance, obtained from examining all model effects, opposed to an estimate of the pure error. If the test is statistically significant (*p* < 0.05), it indicates that the model is not well fitted to predict the response. Referring to Tables S1 and S2, a non-significant value of test lack of fit (*p* > 0.05) relative to the pure error indicates that there is a good correlation between the variables and the process response [51]. The adequate precision (Adeq Precision) value of 32.448 for alpha acids and 57.109 for beta acids determined the signal-to-noise ratio (S/N ratio > 4 is desirable) and confirmed an adequate signal for measurement in the reaction space.

**Figure 5:**
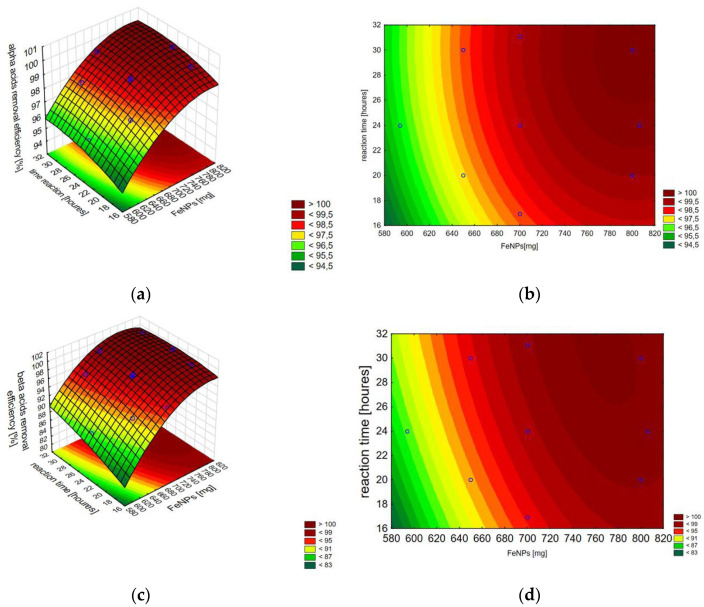
A 3D model surface plot (**a**) and a contour plot (**b**) for alpha acids, and 3D model surface plot (**c**) and a contour plot (**d**) for beta acids. Each point corresponds to a specific combination of factors that were tested in the experiment.

The discussed combined effect of independent variables (IONPs mass and reaction time) on the response value can be presented using a contour plot and a 3D model surface plot (Figure 5), which are a graphical representation of the regression equations for alpha acids and beta acids, respectively:

*Y* = 36.15755 + 0.14589*x*_1_ − 0.00009*x*_1_^2^ + 0.37262*x*_2_ − 0.00620*x*_2_^2^ (4)

*Y* = −131.2640 + 0.5294*x*_1_ − 0.0003*x*_1_^2^ + 1.8280*x*_2_ − 0.0110*x*_2_^2^ − 0.0015*x*_1_*x*_2_ (5)

where *x*_1_ is the mass of IONPs and *x*_2_ is the reaction time. The regression analysis parameters for both acids are provided in Tables S3 and S4.

**Figure 6:**
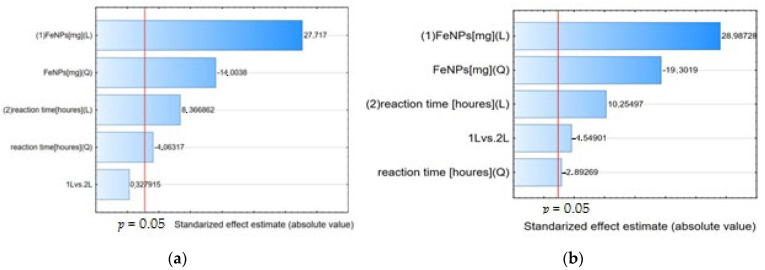
Pareto chart of standardized effects, variable alpha acids (**a**), and beta acids (**b**).

The Pareto chart shows the effect estimates sorted by their absolute value. A Pareto chart of standardized effects (i.e., effects divided by their respective standard deviations) was performed to determine the effect of the studied factors on the alpha acid and beta acid removal efficiency (Figure 6). The vertical line indicates the minimum value of the statistically significant effect, given the model and the set alpha significance level. Both IONPs mass and reaction time had a significant positive effect on the process efficiency, as revealed by the statistically significant *p*-value (*p* < 0.05).

**Figure 7:**
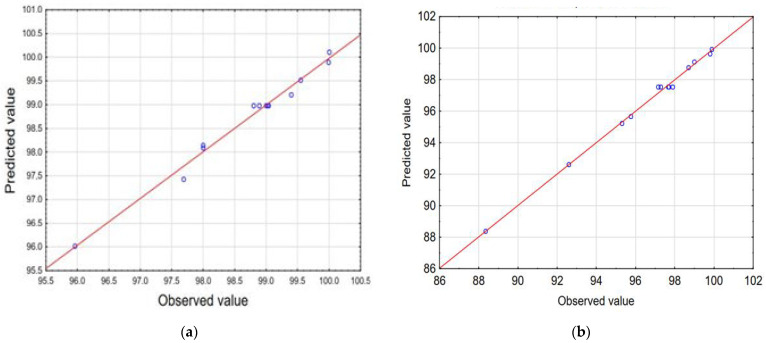
Observed versus predicted values for alpha acid (**a**) and beta acid (**b**) efficiency removal from 100% acetone extract.

The observations indicate a very good correlation between the results obtained in the experiments and the values predicted by the statistical model, which confirms the effectiveness of this model. The scatterplot of the approximated values of the dependent variable against the observed values of this variable is presented in Figure 7.

**Figure 8:**
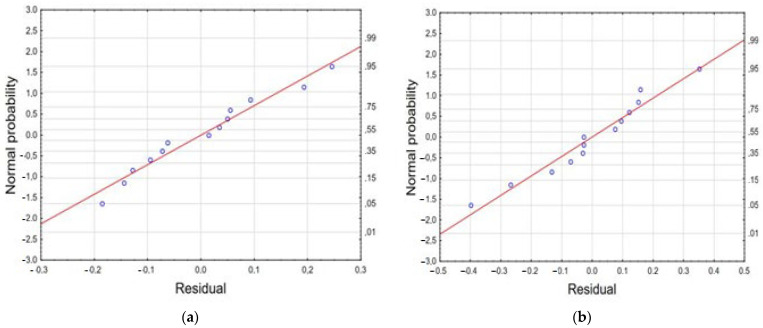
The normal plot of residuals generated for alpha acid (**a**) and beta acid (**b**) removal efficiency.

The normal probability plot is a graphical method for determining the normality of residuals [56,57]. The normal probability plot of residuals versus response (*Y*) is shown in Figure 8. The graphical data on the plot placed at a position close to a straight line shows that the model is sufficient for removing alpha acids and beta acids. The experimental results, model predictions, and response (*Y*), using the Box–Behnken design matrix for the alpha and beta acid removal process, are presented in Tables S5 and S6.

The optimum values of the process variables and the test results under the optimum conditions are shown in Table 8. Accordingly, the alpha acid removal efficiency of 100.11% was determined under the optimum conditions of the independent variables. The maximum efficiency using the confirmatory experiments was 100%. Accordingly, the beta acid removal efficiency of 100.2% was determined under the optimum conditions of the independent variables. The maximum efficiency using the confirmatory experiments was 99.76%. Therefore, there is a good agreement between the predicted and experimental results obtained under the optimum conditions, which confirms the developed model.

#### 3.3.2. Sorption Capacity of IONPs to Alpha and Beta Acids from 50% Acetone Hop Cones Extract

By determining the amount of water added to the extraction medium, it was observed that when adding water to acetone in the amount of 35%, the recovery of xanthohumol reaches 71.229 ± 1.725% after 24 h of contact of 1 mL of extract with 860 mg of nanoparticles. Unfortunately, the purified extract contains traces of beta acids. The most beneficial is the addition of 50% water, which provides about a 60% recovery of xanthohumol. A further increase in the amount of water to 60% causes the loss of about two-thirds of the amount of xanthohumol.

Table 9 shows the actual values of the factors and their corresponding coded levels. According to the RSM, 13 experiments were performed, and their response variable, i.e., alpha and beta acid removal efficiency, was evaluated (Table 10).

**Figure 9:**
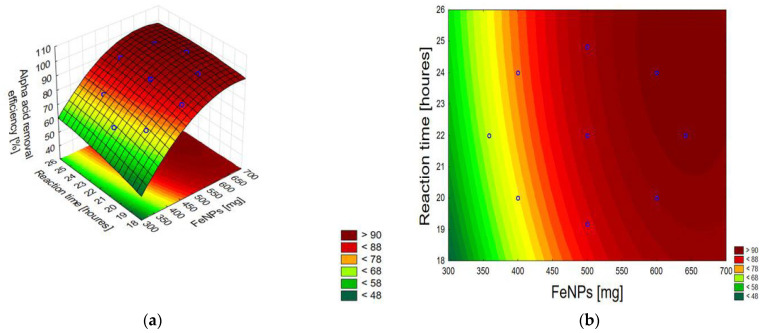

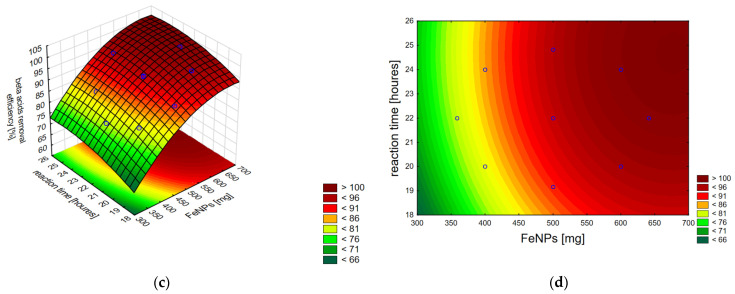
A 3D model surface plot (**a**) and a contour plot (**b**) for alpha acids, and 3D model surface plot (**c**) and a contour plot (**d**) for beta acid removal form 50% acetone extract. Each point corresponds to a specific combination of factors that were tested in the experiment.

The ANOVA analysis of the model suggests that the independent variables (IONPs mass and reaction time) had a significant effect (*p* < 0.05) on the prediction of the efficiency of alpha and beta acid removal from 50% acetone extract (Tables S7 and S8). The R^2^ value for the efficiency of alpha acid removal is 0.9994, and the adjusted R^2^ value is very similar and is 0.9990; the precision value (Adeq precision) is greater than 4 and is 405.508 (Table S7). In turn, the R^2^ value for beta acid removal is 0.9930, and the adjusted R^2^ in this case is 0.9881. The precision value (38.1451) indicates the existence of a desirable relationship between the signal and the measurement in the reaction space. A contour plot and a 3D model surface plots are presented on Figure 9.

The corresponding polynomial equations representing the effect of IONPs mass and reaction time on the efficiency of alpha and beta acid removal from 50% acetone extracts are as follows, respectively:

*Y* = −172.7082 + 0.5282*x*_1_ − 0.0004*x*_1_^2^ + 8.5072*x*_2_ − 0.1339*x*_2_^2^ − 0.0031*x*_1_*x*_2_ (6)

*Y* = −96.5269 + 0.2668*x*_1_ − 0.0002*x*_1_^2^ + 8.7006*x*_2_ − 0.1763*x*_2_^2^ (7)

where *x*_1_ is the mass of IONPs and *x*_2_ is the reaction time.

**Figure 10:**
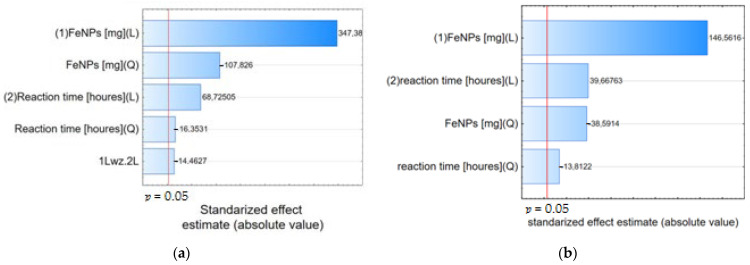
Pareto chart of standardized effects of IONPs mass and reaction time on effectiveness of alpha acid (**a**) and beta acid (**b**) removal from 50% acetone extract.

Regression parameters provided for the polynomial regression model for alpha and beta acids removal from 50% acetone extract are collected in Tables S9 and S10. Pareto charts confirm the significant influence of the studied factors, i.e., IONPs mass and reaction time on the efficiency of alpha and beta acid removal from 50% acetone extract (Figure 10).

**Figure 11:**
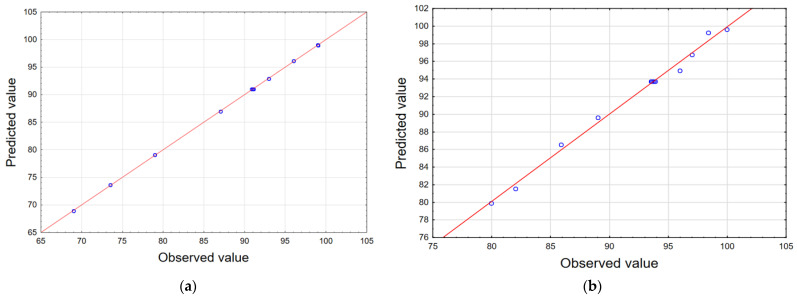
Scatterplot of the approximated values of the dependent variable against the observed values of this variable for alpha (**a**) and beta (**b**) acid removal from 50% acetone extract.

The performed experiments confirm a very good correlation between the results obtained in the experiments and the values predicted by the statistical model (Figure 11).

**Figure 12:**
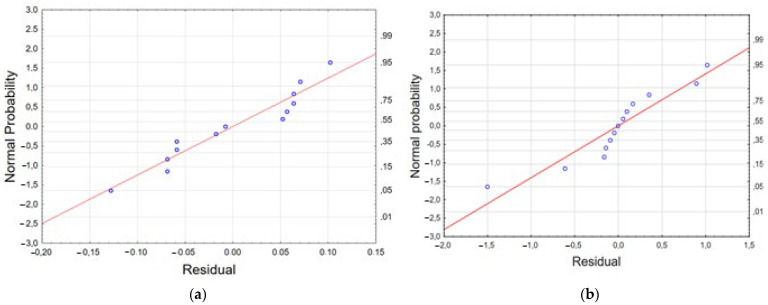
The normal plot of residuals generated for alpha acid (**a**) and beta acid (**b**) removal efficiency from 50% acetone extract.

The experimental results, model predictions, and the response (*Y*) using the Box–Behnken design matrix for the alpha and beta acid removal process are presented in Tables S11 and S12 and Figure 12.

The optimum values of the process variables and the test results under the optimum conditions are shown in Table 11.

### 3.4. The Optimal Conditions for Hop Cones Extracts Purification in the Aim to Xanthohumol Isolation

#### 3.4.1. HPLC

**Figure 13:**
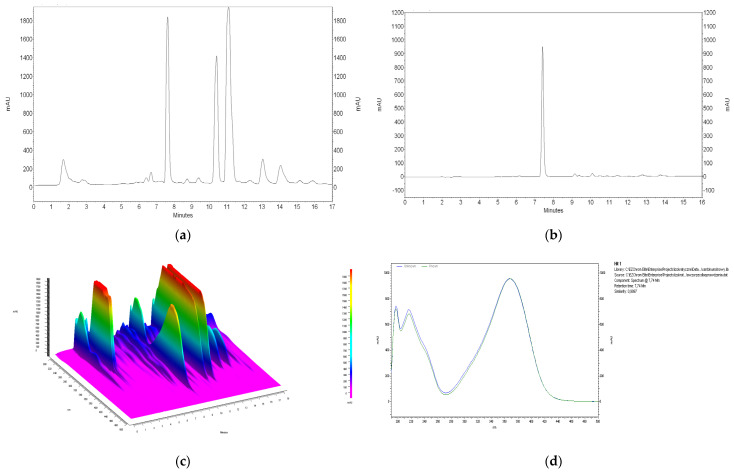
HPLC chromatogram of acetone extract of hop cones “Dary Natury” (**a**), xanthohumol peak isolated from 100% acetone extract after 24 h of contact time of 1 mL of extract with 700 mg of iron oxide nanoparticles (**b**), chromatogram of 3D 100% acetone extract (**c**), overlaid spectrum of xanthohumol standard with spectrum of peak isolated from extract (**d**).

**Figure 14:**
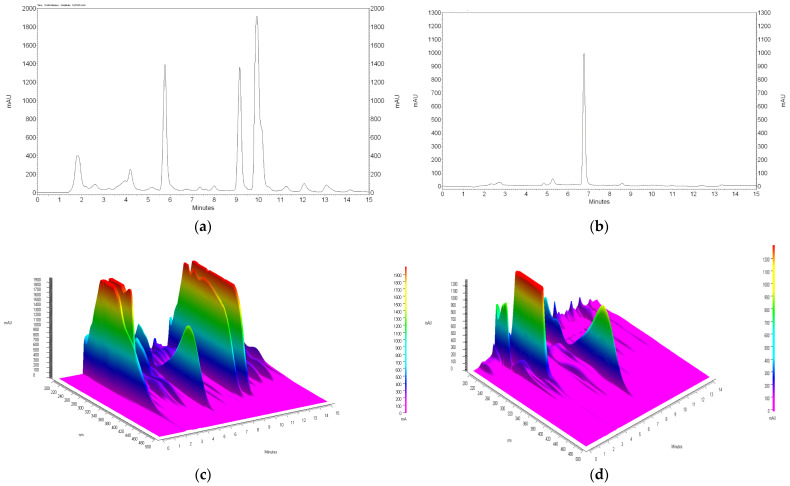
HPLC chromatogram of acetone extract of hop cones “Dary Natury” (**a**), xanthohumol peak isolated from acetone extract after 24 h of contact time of 1 mL of extract with 650 mg of iron oxide nanoparticles (**b**), chromatogram of 3D 50% acetone extract (**c**), chromatogram of 3D 50% acetone extract after contact with nanoparticles (**d**).

**Figure 15:**
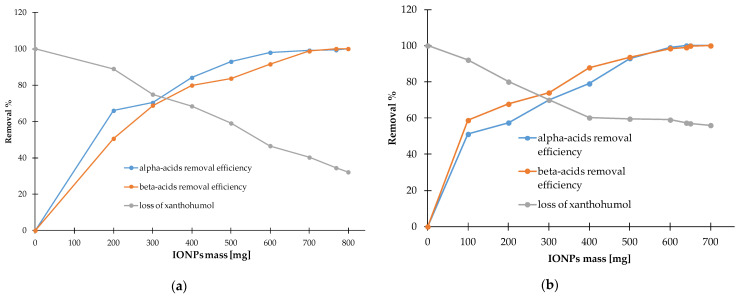
Loss of xanthohumol from the extract along with efficiency of alpha and beta acid removal from 100% acetone (**a**) and 50% acetone (**b**).

Chromatograms showing the isolation of xanthohumol from 100% acetone to 50% acetone extracts under optimized conditions are shown in Figure 13 and Figure 14. It should be noted that the removal of alpha and beta acids from the extracts using IONPs is associated with the loss of xanthohumol from the extract. In the case of 100% acetone extracts, the recovery of xanthohumol is at the level of 40.21 ± 2.81%, which allows obtaining a solution with a concentration of 40.58 ± 2.84 µg mL^−1^, while the use of 50% acetone improves the recovery to 57.11 ± 0.83% and the final product contains xanthohumol with a concentration of 57.64 ± 0.83 µg mL^−1^ in the purified extract. The relationship showing how the increase in the mass of nanoparticles affects the removal of alpha and beta acids and the loss of xanthohumol is presented in Figure 15. As can be seen from this comparison, the use of 50% acetone extract provides more favorable conditions due to the smaller amount of nanoparticles required for extract purification and a higher recovery of xanthohumol.

#### 3.4.2. FT-IR/ATR Analysis

**Figure 16:**
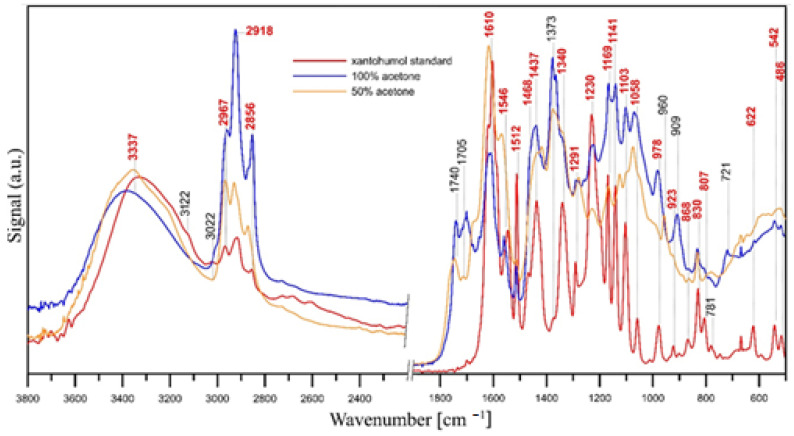
FT-IR/ATR spectra of xanthohumol standard (red line), 100% acetone extract (blue line) and 50% acetone extract (yellow line).

FT-IR/ATR spectroscopy was used confirm the presence and purity of xanthohumol in the extracts. The spectra are shown in Figure 16. The spectral region 2200–1900 cm^−1^ has been cut out in the figure because no significant peaks are observed within this range.

The spectrum of the xanthohumol standard shows characteristic bands for this compound: a broad band of –OH groups connected by inter- and intramolecular hydrogen bonds at 3337 cm^−1^; a band of O-H stretching vibrations and C-H groups in the aromatic ring at 3122 cm^−1^; a band of -C=C- groups vibrations at 3022 cm^−1^, within 1603–1512 cm^−1^ range and at 978 cm^−1^; bands of –CH_3_ groups vibrations in the range of 2967–2856 cm^−1^ and 1468–1373 cm^−1^ (C-H bending vibrations); a band of C=O stretching vibrations at 1619 cm^−1^, a band of –OH groups bending vibrations within 1340–1291 cm^−1^ range; a band of C-O groups vibrations at 1230, 1141, 1103, 1058 cm^−1^; a coupled C–O and O–H vibration band at 1169 cm^−1^; and vibrations of C-H groups in the aromatic ring in the 923–781 cm^−1^ and 622–486 cm^−1^ ranges [58,59,60].

The position and intensity of the bands in the IR spectra of xanthohumol isolated with 100% acetone and 50% acetone (Figure 16) confirm the presence of xanthohumol in the extracts (the bands overlapping with the bands of the xanthohumol standard are marked in red). Additional bands at ca. 1740 cm^−1^ and 1705 cm^−1^ (C=O bands) in the spectra of isolated xanthohumol and higher intensity of bands in the ranges of 2967–2856 cm^−1^ and 1468–1373 cm^−1^ (C-H groups vibration) may indicate the incomplete evaporation of the solvent (acetone) used for extraction. However, bands in the 1740–1700 cm^−1^ range may also indicate the presence of small amounts of other compounds containing C=O groups (i.a. alpha and beta acids) in the extracts and C-H, C-C and C=C groups (C-H deformation and/or C-C skeleton vibrations at 960, 909 and 721 cm^−1^) [61], with these bands having lower intensity in the case of 50% acetone extract. This indicates the higher purity of this extract. The bands of carbonyl groups (C=O) are usually the most intense bands in IR spectra, hence even trace amounts of compounds containing C=O groups will be visible in the spectra.

## 4. Discussion

To extract xanthohumol from hop cones, 50% acetone was applied, which is commonly used for solvent extraction. In order to support the extraction, an ultrasound with a controlled temperature was used to prevent the isomerization of xanthohumol, which under the influence of higher temperatures, changes into a more soluble but less active iso-form. Acoustic cavitation is the main mechanism involved in ultrasound-assisted extraction; therefore, there is no need to increase the temperature to increase the extraction efficiency [62].

HPLC analysis showed that among the three selected hop varieties, i.e., “Marynka”, “Dary Natury”, and “Lubelski”, it was “Dary Natury” that was characterized by the highest content of xanthohumol. Depending on the type of solvent (propanol 80% methanol, methanol-ethyl), the concentration of xanthohumol in the extract ranged from 97 to 121 µg/mL, which, when converted to the mass of the raw material, gives a content of 1.94 mg/g to 2.42 mg/g, or about 0.2%. This is a typical value, because according to previous studies, the content of xanthohumol in Polish hops ranges from 0.1 to 1% [7].

The isolation of xanthohumol from hop extracts usually requires several steps because extracts prepared using organic solvents are not selective for xanthohumol and contain polyphenols, bitter acids, prenylflavonoids, fats, and waxes [26,63]. To purify the extract, semi-preparative scale chromatographic separation using reversed-phase Sephadex LH-20 or C18 columns is usually used [64]. However, this requires the use of additional organic solvents, usually methanol, to elute xanthohumol. Another method is to add 5% sodium carbonate solution in water to the concentrated acetone extract, which usually causes precipitation of chlorophyll and other fatty substances. In this case, the prenylflavonoids are present in the phenolate form and must be recovered in a subsequent extraction, usually with ethyl acetate after acidification. After extraction, xanthohumol is separated in the next step of preparative chromatography [7].

In our proposal, magnetic dispersive extraction was used for the purification of extracts from soft resins that mainly consist of alpha and beta acids by adding iron oxide nanoparticles. Extract purification with nanoparticles can be explained by a phenomenon that takes place during the so-called green synthesis of nanoparticles. In green synthesis, plant extracts are used, due to the content of phytoconstituents, as a reducing and stabilizing agent of nanoparticles. The phytoconstituents adsorbed on the surface stabilize the dispersion and prevent agglomeration [65]. In our study, the prepared nanoparticles in contact with the extract stabilize by the adsorption of the extract components. We noticed that nanoparticles are the most effective in removing (adsorption) alpha acids from all extracts prepared. Most of them are removed from the extracts very quickly, within the first hours. The removal of beta acids can be achieved by extending the contact time of nanoparticles with the extract or by adding more mass of nanoparticles. The difference between alpha and beta acids lies in their solubility. Beta acids are more hydrophobic compared to alpha acids, i.e., they are less soluble than alpha acids and do not undergo isomerization. In addition to differences in solubility, the lack of a tertiary alcohol group in the aromatic ring of beta acids may probably weaken and delay their adsorption on nanoparticles [66].

The addition of water to the acetone extract allows for reducing the nanoparticle mass from 800 mg/1 mL of extract to 680 mg/1 mL of extract and shortening the contact time of the extract with nanoparticles from 30 h to 24 h. The beneficial effect of water addition on the removal of alpha and beta acids can be explained by the reduced solubility in hydro acetone. The weakening of the solvation destabilizes the dispersion and causes their displacement from the solution, facilitating their binding to nanoparticles.

The use of the RSM method to optimize the extract purification process using nanoparticles reduced the number of experiments from less than a hundred to twenty-six. The model was verified by an experiment conducted in optimized conditions. The relative standard error between experimental and projected values was <10% in each case, which confirms the model’s consistency and dependability [48,49,53].

An unfavorable limitation of this method of extract purification using magnetic nanoparticles is the loss of some amount of xanthohumol. Xanthohumol losses are approximately 20% lower in the case of the water/acetone extract compared to pure acetone. However, to completely prevent xanthohumol losses, beta acids should be removed in the first purification step by some other method. Adding IONPs in the next step would allow alpha acids to be removed in a shorter time using a smaller mass of nanoparticles without major xanthohumol losses.

## 5. Conclusions

In this work, a new method of purifying hop cone extracts was developed to isolate xanthohumol using superparamagnetic iron oxide nanoparticles. Purified extracts rich in xanthohumol were obtained by adding nanoparticles to extracts prepared using acetone or aqueous acetone. Among the solvents tested, 50% acetone proved to be the most effective in purifying extracts, especially from alpha and beta acids. The optimization of the purification conditions by the RSM allowed obtaining a pure extract containing from 40.58 µg/mL to 57.64 µg/mL of xanthohumol in one step. The study does not take into account the influence of a temperature up to 50–60 °C, considering the xanthohumol stability, pH changes, and solvents from other classes. Future studies should consider other variables and the possibility of shortening the time needed for extract purification, e.g., by pre-eliminating beta acids, which are more difficult to remove.

## Figures and Tables

**Table 1:** The content of xanthohumol in acetone extracts, prepared from different varieties of hop cones.

Dary Natury			Marynka			Lubelski		
Peak Area	Conc. [µg/mL]	SD	Peak Area	Conc. [µg/mL]	SD	Peak Area	Conc. [µg/mL]	SD
77,179,568	101.07	0.13	75,286,351	98.58	0.30	67,738,289	88.64	0.35

**Table 2:** Levels of the factors tested in Plackett–Burman design for 100% acetone.

Factor	Experimental Value for 100% Acetone Extract		Experimental Value for 50% Acetone Extract	
	Low (−1)	High (+1)	Low (−1)	High (+1)
IONPs [mg]	500	700	400	600
Reaction time [houres]	20	24	20	24

**Table 3:** Plackett–Burman design matrix (in coded level) for alpha acids and beta acid removal in different solvents.

Run Order	IONPs [mg]	Reaction Time [Houres]	Removal Efficiency [%] in 100% Acetone		Removal Efficiency [%] in 50% Acetone	
			Alpha Acids	Beta Acids	Alpha Acids	Beta Acids
1	−1	−1	87.12	80.23	76.03	86.01
2	1	−1	97.01	96.43	95.87	95.99
3	−1	1	90.02	83.02	79.10	89.14
4	1	1	99.05	98.05	98.99	98.32
5	−1	−1	87.12	80.12	76.50	86.44
6	1	−1	99.10	96.10	95.77	95.65
7	−1	1	91.02	82.99	79.02	89.12
8	1	1	99.11	98.12	99.00	98.00

**Table 4:** Analysis of the variance for alpha and beta acid removal in 100% acetone.

Factor	SS Alpha Acids	SS Beta Acids	df Alpha Acids	df Beta Acids	MS Alpha Acids	MS Beta Acids	F Alpha Acids	F Beta Acids	P Alpha Acids	P Beta Acids
(1) IONPs [mg]	190.028	485.785	1	1	190.028	485.785	172.559	4235.630	0.0000	0.0000
(2) reaction time [houres]	9.790	10.811	1	1	9.790	10.811	8.890	94.265	0.0307	0.0002
error	5.506	0.573	5	5	1.101	0.115				
SS tot.	205.324	497.169	7	7						
R^2^	0.9727	0.9988								
R^2^ Adjusted	0.9618	0.9984								

**Table 5:** Analysis of the variance for alpha and beta acid removal in 50% acetone.

Factor	SS Alpha Acids	SS Beta Acids	Df Alpha Acids	df Beta Acids	MS Alpha Acids	MS Beta Acids	F Alpha Acids	F Beta Acids	P Alpha Acids	P Beta Acids
(1) IONPs [mg]	779.730	173.445	1	1	779.730	173.445	20,422.470	2400.544	0.0000	0.0000
(2) reaction time [houres]	17.820	13.755	1	1	17.820	13.755	466.75	190.374	0.0000	0.0000
error	0.191	0.361	5	5	0.038	0.072				
SS tot.	797.741	187.562	7	7	779.730					
R^2^	0.9998	0.9981								
R^2^ Adjusted	0.9997	0.9973								

**Table 6:** Values of the variables and their corresponding coded levels used to design alpha-, and beta- acids removal from 100% acetone extract.

Variable	Coded Values		
	−1 (Low)	0 (Medium)	1 (High)
IONPs (mg)	650	700	800
reaction time (h)	20	24	30

**Table 7:** Central composite design and experimental data obtained for various IONPs masses and times used to alpha-, and beta-acids removal from 100% acetone extract.

Standard Run	IONPs Mass (mg)	Reaction Time (Houres)
1	650	20
2	650	30
3	800	20
4	800	30
5	593.93	24
6	806.07	24
7	700	16.93
8	700	31.07
9 (C)	700	24
10 (C)	700	24
11 (C)	700	24
12 (C)	700	24
13 (C)	700	24

**Table 8:** Optimum value of the process variables for maximum alpha and beta acid removal efficiency and predicted and experimental value for the responses at optimum conditions.

	IONPs [mg]	Reaction Time [Houres]	Removal [%]		
			Predicted	Experimental	RSD%
alpha acids	799.03	29.80	100.11	100.00	0.078
beta acids	768.37	30.70	100.20	99.76	0.311

**Table 9:** Values of the variables and their corresponding coded levels used to design alpha-, and beta- acids removal from 50% acetone extract.

Variable	Coded Values		
	−1 (Low)	0 (Medium)	1 (High)
IONPs (mg)	400	500	600
reaction time (h)	20	22	24

**Table 10:** Central composite design and experimental data obtained for various IONPs masses and times used to alpha-, and beta- acids removal from 50% acetone extract.

Standard Run	IONPs Mass [mg]	Reaction Time [Houres]
1	400	20
2	400	24
3	600	20
4	600	24
5	358.58	22
6	641.42	22
7	500	19.17
8	500	24.83
9 (C)	500	22
10 (C)	500	22
11 (C)	500	22
12 (C)	500	22
13 (C)	500	22

**Table 11:** Optimum value of the process variables for maximum alpha and beta acid removal efficiency from 50% acetone extract and predicted and experimental value for the responses at optimum conditions.

	IONPs [mg]	Reaction Time [Houres]	Removal [%]		
			Predicted	Experimental	RSD%
alpha acids	640.11	24.30	99.65	100.00	0.248
beta acids	677.07	24.70	101.15	99.76	0.978

## Data Availability

The original contributions presented in the study are included in the article/Supplementary Materials, further inquiries can be directed to the corresponding author/s.

## Supplementary Materials

The following supporting information can be downloaded at: https://www.mdpi.com/article/10.3390/ma17194827/s1, Table S1: ANOVA of quadratic response surface model for alpha acids removal from 100% acetone extract; Table S2: ANOVA of quadratic response surface model for beta acids removal from 100% acetone extract; Table S3: Regression parameters provided for the polynomial regression model for alpha acids removal from 100% acetone extract; Table S4: Regression parameters provided for the polynomial regression model for beta acids removal from 100% acetone extract; Table S5: Observed, approximated, and residual values of alpha acids removal efficiency from 100% acetone extract given for each run; Table S6: Observed, approximated, and residual values of beta acids removal efficiency from 100% acetone extract given for each run; Table S7: ANOVA of quadratic response surface model for alpha- acids removal from 50% acetone extract; Table S8: ANOVA of quadratic response surface model for beta- acids removal from 50% acetone extract; Table S9: Regression parameters provided for the polynomial regression model for alpha acids removal from 50% acetone extract; Table S10: Regression parameters provided for the polynomial regression model for beta acids removal from 50% acetone extract; Table S11: Observed, approximated, and residual values of alpha acids removal efficiency from 50% acetone extract given for each run; Table S12: Observed, approximated, and residual values of beta acids removal efficiency from 50% acetone extract given for each run.

## Abbreviations

Adeq precision	adequate precision
6-PN	6-prenylnaringenin
8-PN	8-prenylnaringenin
DAD	diode array detector
As	asymmetry factor
FT-IR/ATR	attenuated total reflectance Fourier-transform infrared
BBD	Box–Behnken design
BET	Brunauer–Emmett–Teller theory
CCD	central composite design
DES	deep eutectic solvents
DMXN	desmethylxanthohumol
FT-IR	Fourier-transform infrared spectroscopy
HPLC	high-performance liquid chromatography
IONPs	iron oxide nanoparticles
IXN	isoxanthohumol
LaDES	lactic acid-based deep eutectic solvents
LOD	limits of detection
LOQ	limits of quantification
MSPE	magnetic dispersive micro-solid phase extraction
PLE	pressurized liquid extraction
R^2^	correlation coefficient
RSD	relative standard deviation
RSM	response surface methodology
S/N	signal-to-noise ratio
tr	retention time
XN	xanthohumol
