# Supplementary material for: Application of Response Surface Methodology to Design and Optimize Purification of Acetone or Aqueous Acetone Extracts of Hop Cones (Humulus lupulus L.) Using Superparamagnetic Iron Oxide Nanoparticles for Xanthohumol Isolation
Source: Materials (Basel). 2024 Sep 30;17(19):4827. doi: 10.3390/ma17194827 (PMC11477801; doi:10.3390/ma17194827)
Supplement: Supplementary file 1 [file materials-17-04827-s001.zip › materials-3209249-supplementary.pdf]

Table S1. ANOVA of quadratic response surface model for alpha acids removal from 100% acetone extract.

Factor	SS	df	MS	F	p
(1)FeNPs(mg)(L)	7.98962	1	7.989616	277.3400	0.000001
FeNPs(mg)(Q)	2.03951	1	2.039512	70.7967	0.000066
(2)Reaction time(houres)(L)	0.72805	1	0.728045	25.2723	0.001518
Reaction time(houres)(Q)	0.17170	1	0.171697	5.9601	0.044672
1L vs.2L	0.00112	1	0.001118	0.0388	0.849407
error	0.20166	7	0.028808		
SStot	14.42397	12			
R- squared	0.9860				
Adjusted R squared	0.9760				
Adequate Precision	32.4498				

Table S2. ANOVA of quadratic response surface model for beta acids removal from 100% acetone extract.

Factor	SS	df	MS	F	p
**(1)FeNPs(mg)(L)**	**54.8859**	**1**	**54.88594**	**840.2626**	**0.000000**
**FeNPs(mg)(Q)**	**24.3359**	**1**	**24.33591**	**372.5646**	**0.000000**
**(2)Reaction time(houres)(L)**	**6.8693**	**1**	**6.86934**	**105.1644**	**0.000018**
**Reaction time(houres)(Q)**	**0.5466**	**1**	**0.54658**	**8.3677**	**0.023227**
**1L vs.2L**	**1.3517**	**1**	**1.35170**	**20.6935**	**0.002639**
**error**	**0.4572**	**7**	**0.06532**		
**SStot**	**122.3720**	**12**			
**R- squared**	**0.9963**				
**Adjusted R squared**	**0.9936**				
**Adequate Precision**	**57.1096**				

Table S3. Regression parameters provided for the polynomial regression model for alpha acids removal from 100% acetone extract.

Factor	Regr. Coeff.	Standard error	t(8)	p	-95%. Cmf. Limt	+95%. Cmf. Lmt
**Mean/Interc.**	**36.15755**	**3.554211**	**10.1732**	**0.000526**	**26.28948**	**46.02562**
**(1)FeNPs(mg)(L)**	**0.14589**	**0.009298**	**15.6904**	**0.000096**	**0.12007**	**0.17171**
**FeNPs(mg)(Q)**	**-0.00009**	**0.000006**	**-14.0400**	**0.000149**	**-0.00011**	**-0.00007**
**(2)Reaction time(houres)(L)**	**0.37262**	**0.074956**	**4.9712**	**0.007645**	**0.16451**	**0.58074**
**Reaction time(houres)(Q)**	**-0.00620**	**0.001516**	**-4.0867**	**0.015016**	**-0.01041**	**-0.00199**

Table S4. Regression parameters provided for the polynomial regression model for beta acids removal from 100% acetone extract.

Factor	Regr.Coeff.	Standard error	t(8)	p	-95%. Cmf. Limt	+95%. Cmf. Lmt
**Mean/Interc.**	**-131.264**	**10.31056**	**-12.7310**	**0.000004**	**-155.645**	**-106.883**
**(1)FeNPs(mg)(L)**	**0.529**	**0.02429**	**21.7954**	**0.000000**	**0.472**	**0.587**
**FeNPs(mg)(Q)**	**-0.000**	**0.00002**	**-19.3019**	**0.000000**	**-0.000**	**-0.000**
**(2)Reaction time(houres)(L)**	**1.828**	**0.29282**	**6.2430**	**0.000427**	**1.136**	**2.520**
**Reaction time(houres)(Q)**	**-0.011**	**0.00381**	**-2.8927**	**0.023227**	**-0.020**	**-0.002**
**1L vs. 2L**	**-0.001**	**0.00033**	**-4.5490**	**0.002639**	**-0.002**	**-0.001**

Table S5. Observed. approximated. and residual values of alpha acids removal efficiency from 100% acetone extract given for each run.

Standard run	FeNPs [mg]	Reaction time [h]	alpha-acids removal efficiency Experimental [%]	Predicted [%]	Residuals
1	650	20	97.69	97.44	0.245792
2	650	30	98.00	98.07	-0.071999
3	800	20	99.55	99.50	0.049852
4	800	30	100.00	100.13	-0.127940
5	593.93	24	95.96	96.02	-0.062379
6	806.07	24	99.99	99.89	0.093170
7	700	16.93	98.00	98.14	-0.143773
8	700	31.07	99.40	99.20	0.193122
9 (C)	700	24	99.00	98.98	0.014831
10 (C)	700	24	98.89	98.98	-0.095169
11 (C)	700	24	99.04	98.98	0.054831
12 (C)	700	24	99.02	98.98	0.034831
13 (C)	700	24	98.80	98.98	-0.185169

Table S6. Observed. approximated. and residual values of beta acids removal efficiency from 100% acetone extract given for each run.

Standard run	FeNPs (mg)	Reaction time (houres)	beta-acids removal efficiency Experimental (%)	Predicted (%)	Residuals
1	650	20	92.60	92.63	-0.028091
2	65	30	95.76	95.66	0.095706
3	800	20	99.00	99.13	-0.132763
4	800	30	99.89	99.92	-0.031292
5	593.93	24	88.35	88.38	-0.027873
6	806.07	24	99.80	99.64	0.158674
7	700	16.9	95.30	95.22	0.075373
8	700	31.01	98.70	98.77	-0.070278
9 (C)	700	24	97.28	97.55	-0.267891
10 (C)	700	24	97.70	97.55	0.152109

11 (C)	700	24	97.90	97.55	0.352109
12 (C)	700	24	97.15	97.55	-0.397891
13 (C)	700	24	97.67	97.55	0.122109

Table S7. ANOVA of quadratic response surface model for alpha- acids removal from 50% acetone extract.

Factor	SS	df	MS	F	p
(1)FeNPs(mg)(L)	901.437	1	901.4375	100820.5	0.000000
FeNPs(mg)(Q)	86.850	1	86.8504	9713.7	0.000000
(2)Reaction time(houres)(L)	35.282	1	35.2818	3946.1	0.000000
Reaction time(houres)(Q)	1.998	1	1.9976	223.4	0.000001
1L vs.2L	1.562	1	1.5625	174.8	0.000003
error	0.063	7	0.0089		
SStot	1025.235	12			
R- squared	0.9994				
Adjusted R squared	0.9990				
Adequate Precision	405.5077				

Table S8. ANOVA of quadratic response surface model for beta- acids removal from 50% acetone extract.

**Factor**	**SS**	**df**	**MS**	**F**	**p**
**(1)FeNPs(mg)(L)**	389.4380	1	389.4380	868.9555	0.000000
**FeNPs(mg)(Q)**	27.0010	1	27.0010	60.2475	0.000110
**(2)Reaction time(houres)(L)**	28.5279	1	28.5279	63.6546	0.000093
**Reaction time(houres)(Q)**	3.4588	1	3.4588	7.7176	0.027375
**1L vs.2L**	1.5500	1	1.5500	3.4586	0.105254
**Error**	3.1372	7	0.4482		
**SStot**	451.0754	12			
**R- squared**	0.9930				
**Adjusted R squared**	0.9881				
**Adequate Precision**	38.1451				

Table S9. Regression parameters provided for the polynomial regression model for alpha acids removal from 50% acetone extract.

Factor	Regr.Coeff.	Standard error	t(8)	p	-95%. Cmf. Limt	+95%. Cmf. Lmt
Mean/Interc.	-172.708	5.225008	-33.0541	0.000000	-185.063	-160.353
(1)FeNPs(mg)(L)	0.528	0.006325	83.5103	0.000000	0.513	0.543
FeNPs(mg)(Q)	-0.000	0.000004	-98.5581	0.000000	-0.000	-0.000
(2)Reaction	8.507	0.412028	20.6470	0.000000	7.533	9.481

time(houres)(L)						
Reaction time(houres)(Q)	-0.134	0.008963	-14.9474	0.000001	-0.155	-0.113
1L vs. 2L	-0.003	0.000236	-13.2196	0.000003	-0.004	-0.003

Table S10. Regression parameters provided for the polynomial regression model for beta acids removal from 50% acetone extract.

Factor	Regr.Coeff.	Standard error	t(8)	p	-95%. Cmf. Limt	+95%. Cmf. Lmt
**Mean/Interc.**	-96.5269	6.453509	-14.9573	0.000116	-114.445	-78.6091
**(1)FeNPs(mg)(L)**	0.2668	0.005127	52.0326	0.000001	0.253	0.2810
**FeNPs(mg)(Q)**	-0.0002	0.000005	-38.5914	0.000003	-0.000	-0.0002
**(2)Reaction time(houres)(L)**	8.7006	0.562063	15.4797	0.000102	7.140	10.2611
**Reaction time(houres)(Q)**	-0.1763	0.012763	-13.8122	0.000159	-0.212	-0.1408

Table S11. Observed. approximated. and residual values of alpha acids removal efficiency from 50% acetone extract given for each run.

Standard run	FeNPs (mg)	Reaction time(houres)	alpha-acids removal efficiency		Residuals
			Experimental (%)	Predicted (%)	
**1**	400	20	73.54000	73.60863	-0.068628
**2**	400	24	78.99000	79.05873	-0.068735
**3**	600	20	96.03000	96.08877	-0.058765
**4**	600	24	98.98000	99.03887	-0.058872
**5**	358.58	22	69.01000	68.93928	0.070724
**6**	641.42	22	99.02000	98.96322	0.056776
**7**	500	19.17	87.04000	86.97633	0.063674
**8**	500	24.83	92.98000	92.91617	0.063826
**9 (C)**	500	22	91.01000	91.01800	-0.008000
**10 (C)**	500	22	91.07000	91.01800	0.052000
**11 (C)**	500	22	91.12000	91.01800	0.102000
**12 (C)**	500	22	91.00000	91.01800	-0.018000
**13 (C)**	500	22	90.89000	91.01800	-0.128000

Table S12. Observed. approximated. and residual values of beta acids removal efficiency from 50% acetone extract given for each run.

Standard run	FeNPs (mg)	Reaction time(houres)	beta-acids removal efficiency		Residuals
			Experimental (%)	Predicted (%)	
**1**	400	20	82.02	82.16	-0.14328
**2**	400	24	85.89	85.94	-0.05005
**3**	600	20	97.01	96.12	0.89255
**4**	600	24	98.39	99.89	-1.50422
**5**	358.58	22	79.95	79.9	0.05334
**6**	641.42	22	99.98	99.63	0.34916
**7**	500	19.17	89.01	89.62	-0.61317
**8**	500	24.83	95.98	94.96	1.01567
**9 (C)**	500	22	93.87	93.70	0.16600
**10 (C)**	500	22	93.70	93.70	-0.00400
**11 (C)**	500	22	93.80	93.70	0.09600
**12 (C)**	500	22	93.54	93.70	-0.16400
**13 (C)**	500	22	93.61	93.70	-0.09400
